# Association of Gait Characteristics and Depression in Patients with Parkinson's Disease Assessed in Goal-Directed Locomotion Task

**DOI:** 10.1155/2017/6434689

**Published:** 2017-02-15

**Authors:** Péter Kincses, Norbert Kovács, Kázmér Karádi, Ádám Feldmann, Krisztina Dorn, Zsuzsanna Aschermann, Sámuel Komoly, Tibor Szolcsányi, Árpád Csathó, János Kállai

**Affiliations:** ^1^Institute of Behavioral Sciences, University of Pécs, Pécs 7624, Hungary; ^2^Clinical Center, Department of Neurology, University of Pécs, Pécs 7623, Hungary; ^3^Pediatric Clinic, Clinical Center, University of Pécs, Pécs 7623, Hungary

## Abstract

*Introduction.* In the genesis of Parkinson's disease (PD) clinical phenomenology the exact nature of the association between bradykinesia and affective variables is unclear. In the present study, we analyzed the gait characteristics and level of depression in PD and healthy volunteers.* Methods.* Patients with PD (*n* = 48) and healthy controls (*n* = 52) were recruited for the present study. Walking speed, stride length, and cadence were compared between groups while participants completed a goal-directed locomotion task under visually controlled (VC) and visually noncontrolled conditions (VnC).* Results.* Significantly higher depression scores were found in PD comparing to healthy control groups. In PD, depression was associated with gait components in the VC wherein the place of the target was visible. In contrast, in healthy subjects the depression was associated with gait components in VnC wherein the location and image of the target were memorized and recalled. In patients with PD and depression, the visually deprived multitask augments the rate of cadence and diminishes stride length, while velocity remains relatively unchanged. The depression associated with gait characteristics as a comorbid affective factor in PD, and that impairs the coherence of gait pattern.* Conclusion.* The relationship between depression and gait parameters appears to indicate that PD not only is a neurological disease but also incorporates affective disturbances that associate with the regulation of gait characteristics.

## 1. Introduction

Studies of gait characteristics in Parkinson's disease (PD) indicate that gait difficulties are commonly associated with affective disturbances [1–3]. In patients with PD, voxel-based morphometric and functional magnetic resonance imaging (fMRI) studies report a correlation between depression and gray matter impairment in the bilateral orbitofrontal cortex, right temporal region, and limbic system [4]. The neural networks involved in gait and balance control include the mesencephalic motor region and the primary and secondary motor cortex [5, 6]. In addition, the basal ganglia and supplementary motor area (SMA), which play a role in the affective organization of goal-directed locomotion, have been implicated in PD [7, 8]. The disruption of these regions induces deficits in action execution and preparation. The NPF's 2012 Parkinson's Outcomes Project [9] found that depression has the greatest impact on gait in patients with PD [7], but the association between depression and gait characteristic can be found in healthy elderly persons as well [10]. Previous studies have demonstrated that the treatment of depression can significantly alleviate motor symptoms. However, patients with PD who receive gait training and other forms of exercise therapy at an early stage demonstrate a significantly slower decline in quality of life than those who received intervention later [9].

In PD, the essential components of gait pattern include stride length, cadence, and velocity [11], wherein studies report decreased stride length and velocity and increased cadence in patients with PD [12]. Many patients with PD experience dysfunctional coordination during walking, and falling is a frequent occurrence [13]. In particular, shorter stride length without surface cues is often detected, and as a compensatory mechanism, this plays an important role in the development of gait hypokinesia [12, 14].

The aim of this study was to examine associations between depression and gait characteristics in patients with PD and healthy elderly control. The present study explores between-group differences in gait pattern and investigates the association between depression and the components of goal-directed locomotion. The authors hypothesized that each examined gait component, velocity, cadence, and stride length, would significantly differ between patients with PD and age-matched healthy controls. In addition, the rate of gait disorder would demonstrate a strong association with depression and quality of life in patients.

## 2. Method

### 2.1. Participants

Patients with PD (*n* = 48) and healthy age and education in years fitted controls (*n* = 52) were recruited for the present study. Patient demographics and clinical characteristics are listed in Table 1. Healthy volunteer participants were recruited from a pensioners' club. Participants were able to walk unsupported and they had normal or corrected vision. All subjects had no history of psychiatric disease, previous treatment with depression, or significant cognitive impairment, were free of acute illness, and did not require nursing care. Patients were recruited from the Outpatients Clinics at the regional Department of Neurology UP Medical School. Considering the required free walk locomotion test condition, patients were only asked for participation who were classified as patients with PD in Hoehn and Yahr stages 3-4 and freezing was not detected [14, 15]. All patients were prescribed levodopa (L-dopa). Cognitive impairment was evaluated using the Mini Mental State Examination (MMSE) [16, 17]. Nondemented persons were only invited to participate in the experimental part of study. In addition, the Montgomery-Åsberg Depression Rating Scale (MADRS) was used to examine affective disturbances [18]. In addition, all subjects were right-handed, as assessed by the Edinburgh Handedness Inventory [19]. Handedness Laterality Quotients were 70% or higher for the right hand in all subjects.

The Unified Parkinson's Disease Rating Scale (UPDRS) [20] was applied to evaluate the PD symptoms (Table 1) and the total UPDRS scores have been used in the statistical analysis. Patients and healthy controls were excluded if they received an MMSE score of less than 23. Following MMSE examination, 40 patients with PD and 49 controls were recruited for experimental analysis. All participants provided written informed consent prior to experimentation. This study was performed in accordance with the Helsinki Declaration and approved by the Regional Research Ethics Committee of the Medical Center in the State University.

### 2.2. Procedure

Global motor function was assessed using the UPDRS by a movement disorder specialist (N.K), when medication was controlled during regular visits to the outpatient clinic. Patients' L-dopa equivalent dosage (LED) was calculated as described by Tomlinson et al. [21]. All patients took their medication in the morning approximately 2 h prior to examination. Participants were invited into a laboratory room and asked to perform two subsequent tasks that were originated from the clinical practice and modelled the everyday locomotion. To assess the gait characteristics, the patients and healthy controls were directed to approach a target in an examination room, where the main gait components of PD were evaluated using operationalized methods. At the beginning of the trial, participants were instructed to stand in front of a visible gray cross target (25 × 25 cm) placed 4 m away on a white wall. They were directed to lift their right arm and to point to a target with their right index finger. Next, while maintaining this arm and finger position, participants were instructed to approach the target and touch it. The goal-directed locomotion test contained two types of multiple task conditions: a simple visual control (VC) and complex nonvisual control (VnC). The first trial was conducted with VC and served as an introductory motor exercise to assess the gait characteristics of participants and to determine stability in circumstances with VC. Following the introductory VC task, three test trials were performed in the VnC condition. During this task, at the start line, after a visual fixation of the target, the participant's eyes were covered by nontransparent swimming glasses and asked to approach and touched the estimated place of the target. The participants were followed up by an assistant. The procedure was safe or falling or other complications did not happen. Under VC and VnC conditions, the participants' gait characteristics, including cadence, stride length, and velocity, were recorded and assessed on a large carpet using a visible scaled ruler. The measurement of gait components was initiated when patients took their first step toward the target and maintained throughout their route towards the goal. Offline video recordings of the route of locomotion were analyzed by two independent raters. Three main gait parameters were assessed, including velocity (m/s), cadence (number of steps from start to target), and stride length (distance in cm between successive heel contact points of the same foot).

### 2.3. Statistical Analysis

SPSS version 22.0 was used for statistical analysis (Chicago, IL, US). For group comparisons, patients with PD and healthy participants were compared. A two-tailed *t*-test for parametric and a Mann–Whitney *U* test for nonparametric data were used to assess between-group differences in age, sex, education level, MADRS, cadence, stride length, and velocity. Kolmogorov-Smirnov tests were used to assess the normality of variables. Logarithmic transformation was applied for cadence data in parametric comparisons. Moreover, Pearson's correlation analyses were used in the control group to assess within-group associations between movement parameters and MADRS data. In addition, in the patients' group, Pearson's correlation analyses were performed to evaluate the relationship between L-dopa dosage and MADRS, disease duration, cadence, stride length, and velocity variables. Gait data differences between groups were analyzed using multivariate test initially (MANCOVA, adjusted for age and gender), with follow-up univariate tests (ANOVA). For multiple comparisons between gait data and depression (following Feldmann et al.'s [4] method MADRS, cutoff = 13 was used) a 2 × 2 × 2 Analysis of Variance (ANOVA) was used with regard to the factors of velocity, cadence, stride length, and MADRS for all dependent variables (two conditions: locomotion with VC and locomotion with VnC) in healthy controls and patients with PD. Post hoc Scheffé-tests were applied to evaluate significant pairwise differences between variables. Reasonable results were listed in post hoc tables only. Levene's test was used to detect differences in the homogeneity of variance. Analyses were two-tailed and significance was set at 0.05.

## 3. Results

### 3.1. The Coherence of Gait Pattern and the Related Affective Associations in Visually Controlled and Visually Noncontrolled Conditions

Correlation analyses in the control group revealed a coherent pattern for velocity, stride length, and cadence. Under both VC and VnC conditions, the association of the three main gait components demonstrated the same coherent configuration (Table 2). During goal-directed locomotion, fast walkers typically exhibit a small cadence and long stride length while small cadence associated with long stride length. In the present task, the depression did not show a significant association with gait pattern under a simple VC conditions; however, in the multitask VnC condition, high depression scores correlated with low velocity, high cadence, and short stride length.

The correlation analysis of patients with PD demonstrated a coherent gait pattern (low velocity, high cadence, and long stride length) similar to that of healthy controls in both the VC and VnC conditions. However, contrary to healthy controls, the depression was linked to low velocity, high cadence, and short stride length in the VC condition. A similar association was not found in the multitask VnC condition between depression and gait parameters. This indicates that the depression associated gait pattern was reverted in the VC and VnC conditions for the control and PD groups. PD-specific UPDRS scores correlated with depression and all gait parameters excluding stride length in the VnC condition. LED values correlated with velocity and stride length only in the VC condition, and disease duration was found to associate with L-dopa dose (Table 3).

### 3.2. Multivariate Analyses and Multiple Comparisons

Comparison of the gait variables in the two groups (PD and healthy controls) showed the presence of significant differences concerning the mean gait scores [MANCOVA Wilks *λ* = .569; *F*(6,88) = 10.083; *p* < 0.001], after controlling for age and gender. Analysis of covariance found significantly lower stride length and higher cadence in patients with PD compared with healthy controls in VC and VnC conditions. The velocity was significantly different in the two groups in the VnC condition only.

### 3.3. Pairwise Results by Scheffé-Test in Cadence

Comparisons of cadence between healthy controls and patients with PD indicated significant differences, wherein patients with PD demonstrated a significant increase in cadence (Table 4). Similar significant results were identified in comparisons of condition (VC × VnC) and with regard to affective disturbance (depression × nondepression). Higher cadence was identified in patients with PD, in the VnC condition, and in participants with a higher depression score. Healthy controls × the VnC condition demonstrated lower cadence compared to patients in the VnC condition. In addition, comparison of healthy controls with depression versus PD patients with depression revealed an increased cadence in PD patients who suffered from depression. Therefore, higher levels of depression and visuospatially deprived examination conditions (VnC) augmented the contrast in cadence between healthy controls and patients with PD, wherein cadence is increased in PD. Further analysis indicated that cadence was increased in the VC condition in patients with PD and depression, compared to the VnC condition. Therefore, patients with PD and higher depression scores demonstrated increased cadence in visuospatially deprived conditions.

### 3.4. Pairwise Results by Scheffé's Test in Stride Length

Differences in stride length between the VC and VnC conditions were significant, wherein stride length was reduced in the visually deprived condition. Patients demonstrated a significantly shorter stride length than healthy controls (Table 4). Participants with elevated depression scores exhibited shorter stride length than participants without indications of depression. In addition, significant differences were identified between nondepressed healthy subjects and nondepressed patients with regard to stride length, wherein the stride length of patients with PD was reduced. Next, stride length was found to decrease in the VnC condition in patients with PD, compared to the VC condition. Therefore, it is possible to suggest that, in patients with PD and depression, the VnC condition significantly decreases stride length.

### 3.5. Pairwise Results by Scheffé's-Test in Velocity

Higher velocity was detected in healthy controls compared to patients with PD. Moreover, lower velocity was identified in PD patients in the VnC condition compared to healthy participants (Table 4).

## 4. Discussion

The main result of this study is that when PD and control participants were tested during goal-directed locomotion high cadence was associated with reduced stride length, while slow walking velocity was associated with high cadence and short stride length in both groups but different in activity rate. When analyzing level of depression in controls, depression did not associate with velocity and other gait components in the common VC condition; however, high levels of depression showed an association with low velocity, high cadence, and short stride length in the multitask VnC condition. In patients, the association of depression and gait components demonstrated a reverted pattern compared to controls. The depression was associated with velocity, stride length, and cadence in VC but not in VnC.

In addition, UPDRS scores were associated with all gait parameters excluding stride length in the VnC condition. The UPDRS, L-dopa equivalent doses, and illness duration showed the conventional syndrome specific associations but need to note that higher L-dopa equivalent doses associated with higher velocity and longer stride length but not with cadence and depression in patients with PD. With regard to intercorrelation analysis of the clinical components of gait, the gait pattern coherence remained intact while participants approached a target in both the VC and VnC conditions. The present results are consistent with the findings of previous studies, indicating that gait patterns are relatively coherent in the early stages of PD compared to healthy participants (low velocity, high cadence, and short stride length). However, in PD, velocity was reduced, cadence was increased, and stride length was shorter than in healthy controls [12]. The reduction in velocity relates to PD-specific visuospatial and proprioceptive impairments [11, 14], in the present case that was significant in complex multiple task in goal-directed locomotion without visual control condition. This indicates that, in patients with PD, depression linked to everyday activities, wherein walking (in a simple VC environment) is a difficult task for the majority of patients. Nevertheless, in the VnC (complex environment without visual guidance) condition, depression does not significantly associate with the goal-directed locomotion of patients with PD. To evaluate this data we need to note that depression-related gait pattern is linked to PD-specific visuospatial ability [22], which is associated with the hemispatial onset of PD symptoms [23], the applied L-dopa equivalent dose [24], disease duration, and current off/on effects [25]. In this study the off/on state had been controlled by the timing of examination, but the hemispatial onset of PD symptoms had not been the object of the present investigation.

It is necessary to establish that, in complex environmental conditions, when participants completed a VnC multitask, gait pattern remained relatively congruent, but patients with PD demonstrated a slower velocity, reduced stride length, and higher cadence compared to healthy controls. The reduction in velocity was significant in the visually deprived condition and in patients with elevated depression. Depression was associated with low velocity, reduced stride length, and elevated cadence in healthy controls in VnC conditions. However, in patients this effect was identified in the VC rather than the VnC condition.

The main results of this study arose from multiple comparisons of gait parameters and affective values between VC and VnC conditions in patients with PD and healthy controls. This analysis indicated that the visuospatially deprived multitask augments the rate of cadence and diminishes stride length in patients with PD and depression, while velocity remains unchanged. These results support previous findings that stride length and cadence are regulated by a bottom-up related reciprocal compensation mechanism [9, 12, 13, 26]. Our results indicate that this compensatory mechanism interacts with depression; however, the depression-related sensitivity of gait components differs. Multivariate analyses demonstrated that depression, first of all in visually noncontrolled multitask condition, produced a general reduction in stride length and increment in cadence in patients with PD to compare healthy controls. Velocity, first of all in visually noncontrolled multitask condition, was lower in patients with PD; however, the influence of depression on this condition has not been detected. It can be supposed that, in these patients, the reduction in the velocity of goal-directed locomotion was likely compensated by higher cadence and shorter stride length.

Lemke et al. [26] reported that depression, Parkinsonian posture, and gait demonstrate a number of similarities. The present evaluation of the relationship between depression and gait parameters appears to indicate that PD not only is a neurological disease but also incorporates a state of affective disturbance that influences the regulation of gait characteristics. In early stage of PD patients, first of all visually deprived multitask situation, depression specially modifies cadence and stride length but has no significant inhibition effect on the velocity of the gait. Consequently, the coherence of the gait pattern gets weaker. The correlation analyses indicated that in common visually controlled condition the depression and gate components showed a correlation pattern in PD (low velocity and stride length and high cadence); however, when multitask was applied in the visually noncontrolled condition the association between depression and gait features disappeared. Limitations to our study include consideration of depression as an aggregated score and its affective characteristic, apathy, quality of life, and the type of cognitive decline had not been detailed. Since the visuospatial disorder and the laterality of the first motor symptoms onset in PD are essential component in the gait control on these domains further research is needed.

## 5. Conclusion

The depression links to gait characteristics as a comorbid affective factor in PD. The findings reported above serve as a laboratory-based demonstration of gait specificity in PD and revealed depression-related dissimilarities in healthy and Parkinsonian gait patterns. Considering the associations between gait and depression these data might aid the development of exact clinical diagnoses, to conduct more efficient rehabilitation training for gait disturbances.

## Figures and Tables

**Table 1:** Demographic and clinical characteristics of Parkinson's disease patients and healthy control persons. MEMS = Mini-Exam of Mental Status; MADRS = Montgomery-Åsberg Depression Rating Scale; VC = visually controlled condition; VnC = visually noncontrolled condition; UPDRS = Unified Parkinson's Disease Rating Scale; LEDD = levodopa equivalent daily dose.

	Parkinson's disease (*N* = 40)	Healthy control (*N* = 49)	*t*; *Z*
			*p* value
Gender, males/females	24/16	24/25	1.0 ns.
Age, mean ± SD, Min–Max	68.0 ± 8.1; 57–80	65.6 ± 5.6; 56–79	1.6 ns.
MEMS (in %) mean ± SD	91.0 ± 16.6	89.4 ± 10.5	0.6 ns.
MADRS total, mean ± SD	18.1 ± 9.1	11.6 ± 8.2	3.5^*∗∗∗*^
Cadence VC ( for 4 m)	9.9 ± 3.5	8.0 ± 2.2	3.1^*∗∗*^
Cadence VnC (for 4 m)	15.1 ± 6.9	9.8 ± 2.6	4.9^*∗∗∗*^
Step length VC (cm)	38.8 ± 11.1	45.9 ± 10.5	3.1^*∗∗*^
Step length VnC (cm)	28.1 ± 9.6	41.0 ± 10.8	5.9^*∗∗∗*^
Velocity VC (m/s)	0.6 ± 0.2	0.7 ± 2.3	1.2 ns.
Velocity VnC (m/s)	0.5 ± 0.2	0.6 ± 0.2	4.0^*∗∗∗*^
Disease duration, mean ± SD, Min–Max	6.7 ± 4.5; (1–16)	N/D	
UPDRS total mean ± SD, Min–Max	58.7 ± 25.5 (28–122)	N/D	
Mentation, mean ± SD	12.1 ± 6.2	N/D	
Daily activities mean ± SD	11.7 ± 7.5	N/D	
Motor examination mean ± SD	31.3 ± 13.7	N/D	
Complication mean ± SD	4.0 ± 3.4	N/D	
LEDD (mg) mean ± SD, Min–Max	712.2 ± 464.5 (154–1880)	N/D	

*p* < 0.01^*∗∗*^; *p* < 0.001^*∗∗∗*^.

**Table 2:** Pearson's correlation of gait characteristics and affective scales in the healthy control group in two conditions (VC = visually controlled condition; VnC = visually noncontrolled condition), MADRS (Montgomery-Åsberg Depression Rating Scale).

	(1)	(2)	(3)	(4)	(5)	(6)	(7)
(1) Velocity VC		−.84^*∗∗∗*^	.87^*∗∗∗*^	.80^*∗∗∗*^	−.61^*∗∗∗*^	.71^*∗∗∗*^	−.21
(2) Cadence VC			−.94^*∗∗∗*^	−.64^*∗∗∗*^	.79^*∗∗∗*^	−.66^*∗∗∗*^	.27
(3) Stride length VC				.72^*∗∗∗*^	−.78^*∗∗∗*^	.75^*∗∗∗*^	−.21
(4) Velocity VnC					−.73^*∗∗∗*^	.78^*∗∗∗*^	−.32^*∗*^
(5) Cadence VnC						−.86^*∗∗∗*^	.39^*∗∗*^
(6) Stride length VnC							−.31^*∗∗*^
(7) MADRS							

*p* < 0.05^*∗*^; *p* < 0.01^*∗∗*^; *p* < 0.001^*∗∗∗*^.

**Table 3:** Persons' correlation of gait characteristics and depression scale in Parkinson's disease group in two conditions (VC = visually controlled condition; VnC = visually noncontrolled condition): MADRS (Montgomery-Åsberg Depression Rating Scale); UPDRS (Unified Parkinson's Disease Rating Scale); LED values; duration of disease.

	(1)	(2)	(3)	(4)	(5)	(6)	(7)	(8)	(9)	(10)
(1) Velocity VC		−.86^*∗∗∗*^	.92^*∗∗∗*^	.75^*∗∗∗*^	−.74^*∗∗*^	.21	−.34^*∗*^	−.44^*∗∗*^	.34^*∗∗*^	.21
(2) Cadence VC			−.91^*∗∗∗*^	−.73^*∗∗*^	.82^*∗∗∗*^	−.20	.33^*∗*^	.37^*∗∗*^	−.27	−.24
(3) Stride length VC				−.75^*∗∗∗*^	−.79^*∗∗∗*^	.16	−.31^*∗*^	−.36^*∗*^	.34^*∗*^	.22
(4) Velocity VnC					−.85^*∗∗∗*^	.42^*∗∗*^	−.25	−.34^*∗*^	.18	.16
(5) Cadence VnC						−.34^*∗*^	.25	.38^*∗∗*^	−.14	−.16
(6) Stride length VnC							−.13	−.15	.04	.08
(7) MADRS								.47^*∗∗*^	−.28	−.21
(8) UPDRS									−.03	.09
(9) LED values										.63^*∗∗∗*^
(10) Duration										

*p* < 0.05^*∗*^; *p* < 0.01^*∗∗*^; *p* < 0.001^*∗∗∗*^.

**Table 4:** Multiple group comparison between patient x control, visually x nonvisually controlled conditions, and depression x nondepression groups with several gait parameters. He = healthy control condition, PD = Patient's condition, VC = visually controlled condition, VnC = visually noncontrolled condition, NoD = nondepressive condition, and DE = depressive condition.

Gait parameters	Mean (SD)						ANOVA intercept	Group comparisons	Scheffé post hoc *p*
	He	PD	VC	VnC	NoD	DE			
Cadence	8.9 (2.5)	12.5 (6.0)	8.86 (3.0)	12.7 (5.6)	9.07 (2.8)	11.08 (5.7)	*F* = 904.14 df = 1; *p* < 0.001	He versus PD	<0.001
								VC versus VnC	<0.001
								NoD versus DE	<0.001
								He × VnC versus PD × VnC	<0.001
								He × DE versus PD × DE	<0.004
								PD × VC × DE versus PD × VnC × DE	<0.006

Stride length (cm)	43,5 (10.8)	33.4 (11.6)	42,8 (11,2)	35,2 (12,1)	42.7 (11.3)	35.6 (12.1)	*F* = 1813 df = 1; *p* < 0.001	He versus PD	<0.001
								VC versus VnC	<0.001
								NoD versus DE	<0.001
								He × NoD versus PD × NoD	<0.005
								PD × VC × DE versus PD × VnC × DE	<0.047

Velocity (m/s)	0.65 (1.44)	0.54 (0.39)	0.65 (0.45)	0.53 (0.74)	0.59 (1.61)	0.40 (0.42)	*F* = 185,48 df = 1; *p* < 0.001	He versus PD	<0.001
								He × VnC versus PD × VnC	<0.048

## References

[1] Cummings J. L. (1992). Depression and parkinson's disease: a review. *American Journal of Psychiatry*.

[2] Paleacu D., Shutzman A., Giladi N., Herman T., Simon E. S., Hausdorff J. M. (2007). Effects of pharmacological therapy on gait and cognitive function in depressed patients. *Clinical Neuropharmacology*.

[3] Chaudhuri K. R., Odin P., Antonini A., Martinez-Martin P. (2011). Parkinson's disease: the non-motor issues. *Parkinsonism & Related Disorders*.

[4] Feldmann A., Illes Z., Kosztolanyi P. (2008). Morphometric changes of gray matter in Parkinson's disease with depression: a voxel-based morphometry study. *Movement Disorders*.

[5] Ziemssen T., Reichmann H. (2007). Non-motor dysfunction in Parkinson's disease. *Parkinsonism and Related Disorders*.

[6] Grabli D., Karachi C., Welter M.-L. (2012). Normal and pathological gait: what we learn from Parkinson's disease. *Journal of Neurology, Neurosurgery and Psychiatry*.

[7] Lord S., Galna B., Coleman S., Burn D., Rochester L. (2013). Mild depressive symptoms are associated with gait impairment in early Parkinson's disease. *Movement Disorders*.

[8] Rocha P. A., Porfírio G. M., Ferraz H. B., Trevisani V. F. M. (2014). Effects of external cues on gait parameters of Parkinson's disease patients: a systematic review. *Clinical Neurology and Neurosurgery*.

[9] Almeida L., Okun M. S., Bowers D. (2015). Prevalence of depression in atypical Parkinsonian disorders versus Parkinson's Disease. *Movement Disorders*.

[10] Brandler T. C., Wang C., Oh-Park M., Holtzer R., Verghese J. (2012). Depressive symptoms and gait dysfunction in the elderly. *American Journal of Geriatric Psychiatry*.

[11] Williams A. J., Peterson D. S., Earhart G. M. (2013). Gait coordination in Parkinson disease: effects of step length and cadence manipulations. *Gait and Posture*.

[12] Morris M. E., Iansek R., Matyas T. A., Summers J. J. (1996). Stride length regulation in Parkinson's disease: normalization strategies and underlying mechanisms. *Brain*.

[13] Nieuwboer A., Dom R., De Weerdt W., Desloovere K., Fieuws S., Broens-Kaucsik E. (2001). Abnormalities of the spatiotemporal characteristics of Gait at the onset of freezing in Parkinson's disease. *Movement Disorders*.

[14] Azulay J.-P., Mesure S., Blin O. (2006). Influence of visual cues on gait in Parkinson's disease: contribution to attention or sensory dependence?. *Journal of the Neurological Sciences*.

[15] Goetz C. G., Poewe W., Rascol O. (2004). Movement Disorder Society Task Force report on the Hoehn and Yahr staging scale: status and recommendations. *Movement Disorders*.

[16] Folstein M. F., Folstein S. E., McHugh P. R. (1975). ‘Mini-mental state’: a practical method for grading the cognitive state of patients for the clinician. *Journal of Psychiatric Research*.

[17] Feher E. P., Mahurin R. K., Doody R. S., Cooke N., Sims J., Pirozzolo F. J. (1992). Establishing the Limits of the Mini-Mental State: examination of ‘Subtests’. *Archives of Neurology*.

[18] Montgomery S. A., Asberg M. (1979). A new depression scale designed to be sensitive to change. *British Journal of Psychiatry*.

[19] Oldfield R. C. (1971). The assessment and analysis of handedness: the Edinburgh inventory. *Neuropsychologia*.

[20] Fahn S., Elton R., UPDRS Program Members, Fahn S., Marsden C., Goldstein M., Calne D. (1987). Unified Parkinson's disease rating scale. *Recent Developments in Parkinson's Disease*.

[21] Tomlinson C. L., Stowe R., Patel S., Rick C., Gray R., Clarke C. E. (2010). Systematic review of levodopa dose equivalency reporting in Parkinson's disease. *Movement Disorders*.

[22] Stuart S., Lord S., Hill E., Rochester L. (2016). Gait in Parkinson's disease: a visuo-cognitive challenge. *Neuroscience and Biobehavioral Reviews*.

[23] Karádi K., Lucza T., Aschermann Z. (2015). Visuospatial impairment in Parkinson’s disease: the role of laterality. *Laterality*.

[24] Fleury V., Cousin E., Czernecki V. (2014). Dopaminergic modulation of emotional conflict in Parkinson's disease. *Frontiers in Aging Neuroscience*.

[25] Moore S. T., MacDougall H. G., Gracies J.-M., Cohen H. S., Ondo W. G. (2007). Long-term monitoring of gait in Parkinson's disease. *Gait and Posture*.

[26] Lemke M. R., Wendorff T., Mieth B., Buhl K., Linnemann M. (2000). Spatiotemporal gait patterns during over ground locomotion in major depression compared with healthy controls. *Journal of Psychiatric Research*.

